# Validation of a convolutional neural network for the automated creation of curved planar reconstruction images along the main pancreatic duct

**DOI:** 10.1007/s11604-022-01339-1

**Published:** 2022-09-19

**Authors:** Yuji Koretsune, Miyuki Sone, Shunsuke Sugawara, Yusuke Wakatsuki, Toshihiro Ishihara, Chihiro Hattori, Yasuko Fujisawa, Masahiko Kusumoto

**Affiliations:** 1 Department of Diagnostic and Interventional Radiology, Osaka University, 2-15 Yamadaoka, Suita, Osaka 565-0871 Japan; 2 Department of Diagnostic Radiology, National Cancer Center Hospital, Chuo City, Japan; 3 Department of Diagnostic Technology, National Cancer Center Hospital, Chuo City, Japan; 4 Canon Medical Systems Corp., Otawara, Japan

**Keywords:** Curved planar reconstruction, Main pancreatic duct, Imaging algorithm, Pancreatic cancer, Deep learning

## Abstract

**Purpose:**

To evaluate the accuracy and time-efficiency of newly developed software in automatically creating curved planar reconstruction (CPR) images along the main pancreatic duct (MPD), which was developed based on a 3-dimensional convolutional neural network, and compare them with those of conventional manually generated CPR ones.

**Materials and methods:**

A total of 100 consecutive patients with MPD dilatation (≥ 3 mm) who underwent contrast-enhanced computed tomography between February 2021 and July 2021 were included in the study. Two radiologists independently performed blinded qualitative analysis of automated and manually created CPR images. They rated overall image quality based on a four-point scale and weighted κ analysis was employed to compare between manually created and automated CPR images. A quantitative analysis of the time required to create CPR images and the total length of the MPD measured from CPR images was performed.

**Results:**

The *κ* value was 0.796, and a good correlation was found between the manually created and automated CPR images. The average time to create automated and manually created CPR images was 61.7 s and 174.6 s, respectively (*P* < 0.001). The total MPD length of the automated and manually created CPR images was 110.5 and 115.6 mm, respectively (*P* = 0.059).

**Conclusion:**

The automated CPR software significantly reduced reconstruction time without compromising image quality.

## Introduction

Pancreatic cancer remains a malignancy with a poor prognosis despite developments in diagnosis and therapeutics. Only 15–20% of patients diagnosed with pancreatic cancer were considered to be surgically “resectable” [1]. Computed tomography (CT) is the imaging modality normally used to evaluate patients with suspected pancreatic cancer. Therefore, precise detection and staging of pancreatic cancer is required from CT scans. Abrupt change or disruption of the main pancreatic duct (MPD) is an essential finding for detecting pancreatic cancer [2]. Curved planar reconstruction (CPR) images delineate the curved anatomical structure including the MPD and display the whole course in a two-dimensional single image. CPR images along the MPD have been reported to be useful for establishing the diagnosis and management of pancreatic diseases [3]. They can be considered supplements for transverse images that could enhance rapid communication of related findings with referring physicians [3, 4]. In addition, CPR images were found to be valuable for predicting vascular invasion and pancreatic cancer resectability [5, 6]. However, CPR images are not widely available because creating high-quality versions requires the expertise of experienced personnel. Moreover, performing CPR manually is time consuming, as it requires placing multiple cursors along the MPD.

In the last decade, artificial intelligence has gradually been adopted in medical imaging, and automatic image processing software has been developed. The development of automatic software for the creation of CPR images along the MPD would allow for the availability of accurate and reliable CPR images globally and might enhance the detectability of pancreatic cancer.

New algorithms to locate the MPD and create CPR images automatically have been previously described [7]. We hypothesized that automated CPR images could be comparable to the quality of manually created CPR images by experienced individuals, in which they require a shorter time to be generated. In this study, we evaluated the accuracy and time efficiency of the image processing software and compared them to those of manually created CPR images.

## Materials and methods

This study was approved by the Institutional Ethics Committee. Informed consent for participation in this study was waived owing to its retrospective nature.

### Patient dataset

**Fig. 1:**
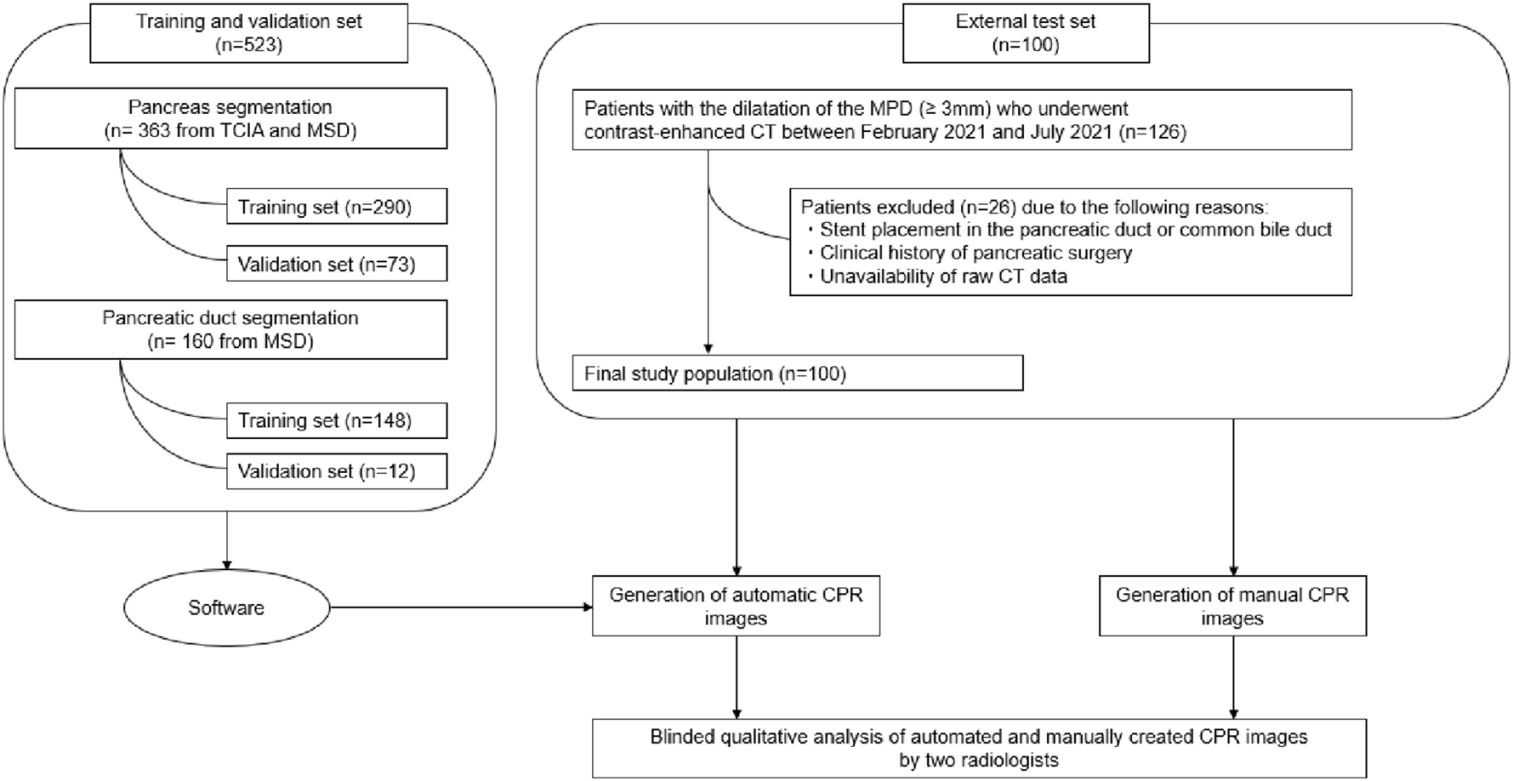
Overview of the study design and patient dataset. *TCIA* The Cancer Imaging Archive Public Access, *MSD* medical segmentation decathlon, *MPD* main pancreatic duct

Overview of the study design and patient datasets are shown in Fig. 1.

Regarding the external test set, a total of 126 consecutive patients who underwent high-spatial-resolution CT (HSR CT) in our institution between February 2021 and July 2021 were identified. Out of these, 100 patients were included in the study. The inclusion criteria were as follows: (a) MPD dilatation ≥ 3 mm and (b) age ≥ 18 years. The exclusion criteria were as follows: (a) stent placement in the pancreatic duct or common bile duct, (b) clinical history of pancreatic surgery, and (c) unavailability of raw CT data (Fig. 1).

Prior to the external test, data of 523 patients were extracted for the training and validation sets from the following public datasets: The Cancer Imaging Archive (TCIA) and Medical Segmentation Decathlon (MSD), which include data on pancreatic cancers [8, 9].

### CT image acquisition and image reconstruction

Most of the CT images (*n* = 94) were acquired using the super-high-resolution mode of the HSR CT scanner (Aquilion Precision; Canon Medical Systems, Otawara, Japan). The parameters employed were as follows: 1792 channels, 0.25 mm × 160 rows detector; slice thickness, 1 mm; pitch factor, 0.569; gantry rotation period, 0.5 s; matrix size, 1024 × 1024 pixels; X-ray voltage, 120 kVp; tube current optimized with automatic exposure control (Volume EC, Canon Medical Systems) using a noise level (standard deviation [SD]) of 12-HU with a thickness of 5.0 mm and a maximum value of 310 mA; and arbitrary field of view.

Some CT images were acquired using another CT scanner (Aquilion ONE (*n* = 1), Aquilion PRIME (*n* = 5); Canon Medical Systems, Otawara, Japan). The parameters employed were as follows: 896 channels; 0.5 mm × 80 rows detector; slice thickness, 1 mm; pitch factor, 0.813; gantry rotation period, 0.5 s; matrix size, 512 × 512 pixels; X-ray voltage, 120 kVp; tube current optimized with automatic exposure control (Volume EC, Canon Medical Systems) using a noise level (SD) of 12-HU with a thickness of 5.0 mm and a maximum value of 310 mA; and arbitrary field of view.

The contrast-enhanced CT images were obtained as follows: The first scan using the bolus-tracking method was performed when the region of interest in the aorta achieved 150-HU. The scans were then performed 20 s and 45 s following the first scan, which were equivalent to the pancreatic and portal venous phases, respectively. The last scan was performed 180 s after contrast agent administration. The contrast material (600 mg of iodine per kilogram of total body weight) was injected for 30 s with a 20-or 22-gauge needle using a power injector (Dual shot; Nemoto Kyorindo, Tokyo, Japan). CT images were reconstructed with hybrid-IR (Adaptive Iterative Dose Reduction 3-Dimensional (3D) [AIDR3D, standard setting]; Canon Medical Systems) or deep learning reconstruction (Advanced Intelligent Clear-IQ Engine [AiCE, body sharp, mild]; Canon Medical Systems). AiCE incorporates deep convolutional neural networks into the reconstruction flow, leading to higher overall image quality and lower image noise compared with hybrid- and model-based iterative reconstruction [10, 11].

### Generation of manual CPR images

**Fig. 2:**
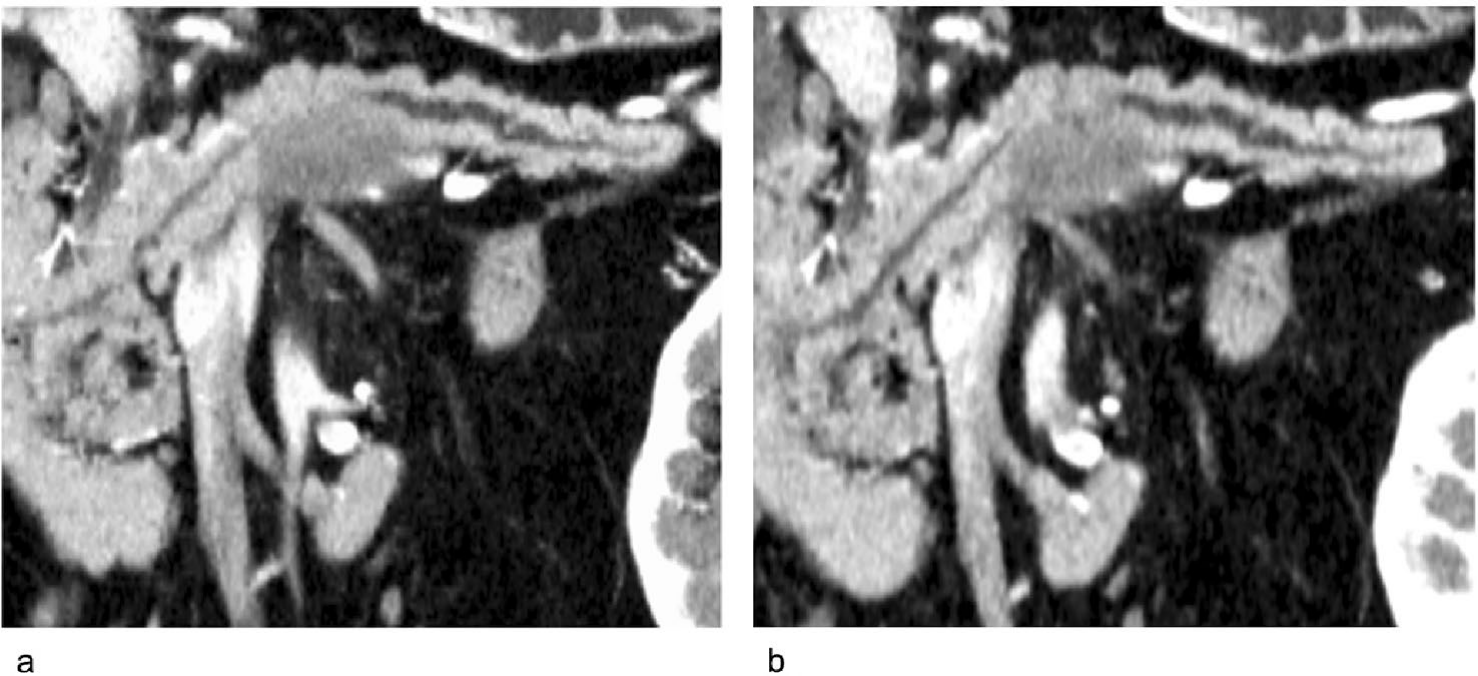
Examples of CPR images obtained from the same clinical case of pancreatic cancer. **a** Manual CPR image created by radiology technicians. **b** Automated CPR image created by our newly developed software. *CPR* curved planar reconstruction

CPR images were obtained based on the pancreatic phase images, which were obtained 20 s following the first scan. Manually created CPR images were generated by one of 10 radiology technicians from our institution who were experienced in the task (at least 30 cases). Images were generated on the operator console of the CT system. They traced the pancreatic duct centerline in the axial image, reconstructed it, and finally generated a single CPR image along the MPD (Fig. 2).

### Generation of automatic CPR images

**Fig. 3:**
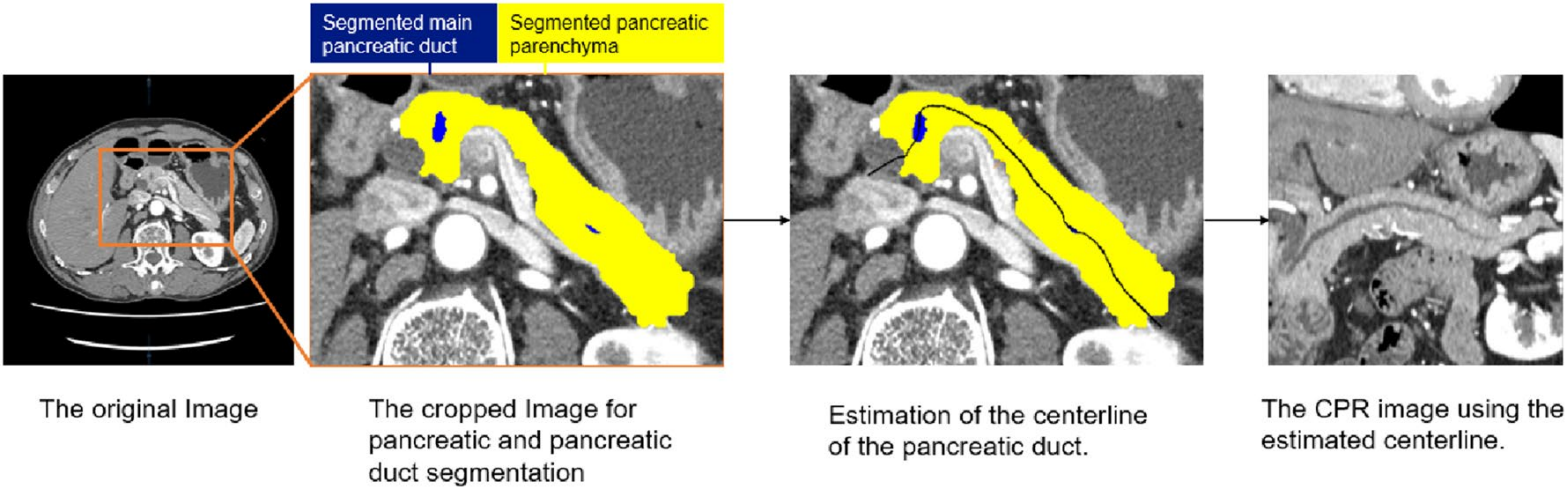
Graphic flowchart displaying the automated process for CPR image generation. *CPR* curved planar reconstruction

The automatic CPR images were created using software under development (Fig. 3), which encompasses three processes: pancreas segmentation, pancreatic duct segmentation, and pancreatic duct centerline detection.

The whole pancreatic region was segmented by a 3D convolutional neural network (3D-CNN) based on U-net. A total of 290 cases from the TCIA and MSD were used for training. In addition, 73 cases from the TCIA and MSD were used for validation of pancreas segmentation.

Pancreatic duct segmentation was also performed using 3D-CNN based on U-net: 148 cases from the MSD were used for training. Furthermore, 12 cases from the MSD were used for validation of pancreatic duct segmentation.

The pancreatic duct centerline was detected by conventional image processing. The optimal pathway was searched from the pancreatic head to tail by employing Dijkstra’s routing algorithm, followed by the identification of the pancreatic duct centerline. The pancreatic duct centerline was defined as follows: (a) it passes through the segmented pancreatic duct, or (b) passes approximately through the center of the segmented pancreas if the MPD was undetectable in pancreatic duct segmentation.

The details of an algorithm have been described previously [7].

Automatic CPR images were generated on a workstation with Intel Xeon Gold 6128 CPU, 128 GB RAM and NVIDIA Quadro RTX 5000 GPU.

### Subjective image analysis

Two radiologists (Y.K. and S.S. with 7 and 19 years of experience, respectively) independently performed a blinded qualitative analysis of the automated and manual CPR images. Prior to evaluation, they received instructions and were trained by six CPR images not included in the study. They evaluated overall image quality based on a four-point scale, where 1: not evaluable due to marked distortion of spatial resolution; 2: poor due to poor visibility of the MPD; 3: fair due to average visibility of the MPD; and 4: good visibility of the MPD. In case of disagreement, the final score was determined by consensus. In regard to the cases where the image quality score of automated CPR images was inferior to that of manually-created ones, the cause was investigated after the image quality assessment.

### Objective image analysis

An objective analysis was performed by one radiologist (Y.K.). The total length of the MPD in the CPR image was measured by placing the cursor along the MPD. In cases where the MPD was divided into multiple segments owing to tumors, the sum of all segments was considered to be the total length. Reconstruction time for the CPR images, measured from the beginning of a working process to the time a CPR image was displayed, was also recorded.

### Statistical analysis

Statistical analysis was performed using commercially available software (SPSS version 27; IBM Corp., Armonk, NY, USA). Weighted *κ* analysis was employed to evaluate the inter-observer agreement and compare the automated and manually created CPR images. The *κ* value was classified as poor (0.0 ≤ *κ* ≤ 0.2), fair (0.2 < *κ* ≤ 0.4), moderate (0.4 < *κ* ≤ 0.6), good (0.6 < *κ* ≤ 0.8), or excellent (0.8 < *κ* ≤ 1.0) [12]. Differences in generation time for CPR images and total length of the MPD described in CPR images were analyzed using paired *t*-tests. *P* < 0.05 was considered statistically significant.

A sample size of 100 patients was calculated so that the lower limit of the *κ* value in 95% confidence interval (CI) exceeded 0.6. Twenty additional patients, not included in the study, were used to calculate the sample size.

## Results

### Patient characteristics

The study population comprised 100 patients (52 men and 48 women) with a median age of 72 years (range: 38–93 years). The median diameter of the maximum MPD was 5 mm (range: 3–13 mm). The main cause of MPD dilation was pancreatic cancer (83%).

The demographic and clinical characteristics of the patients are summarized in Table 1.

**Table 1:** Demographic and clinical characteristics of the patients

Characteristics	Number of patients (*N* = 100)
Age, years	
Median (range)	72 (38–93)
Sex	
Male	52 (52%)
Female	48 (48%)
Diagnosis	
Pancreatic cancer	83 (83%)
Intraductal papillary mucinous neoplasm	9 (9%)
Unknown	7 (7%)
Neuroendocrine carcinoma	1 (1%)
Maximum diameter of the main pancreatic duct (mm)	
Median (range)	5 (3–13)

### CPR image quality

The correspondence rate of the image quality score of the automated and manually created CPR groups was fairly good, with a *κ* value of 0.796 (95% CI 0.649–0.942). Similarly, an inter-observer agreement was good, with a *κ* value of 0.758 (95% CI 0.657–0.858).

Table 2 shows the results of overall image quality based on the four-point scale. Although the proportion of grade 4 (good) appeared slightly higher in the manual CPR group, the difference was not statistically significant (*P* = 0.096).

**Table 2:** Subjective image quality scores for automated and manually created CPR images

	1	2	3	4	Mean	*P*
Grade (*n* = 100)						
Automated CPR images	0	2	22	76	3.72	0.096
Manual CPR images	0	2	17	81	3.78	

Grade 1: not evaluable, Grade 2: poor, Grade 3: fair, Grade 4: good

*CPR* curved planar reconstruction

There were five cases in which the image quality of the automated CPR images was inferior to that of the manually created CPR images. The low score was caused by the undetectability of non-dilated MPD, with a median diameter of 1.85 mm (range: 1.5–2 mm), which was measured on the axial images and manually created CPR images. The pancreatic lesion in which the MPD was not detected in the automated CPR images included pancreatic head in two cases, pancreatic body in one case, and pancreatic tail in two cases.

### Generation time for CPR images

**Fig. 4:**
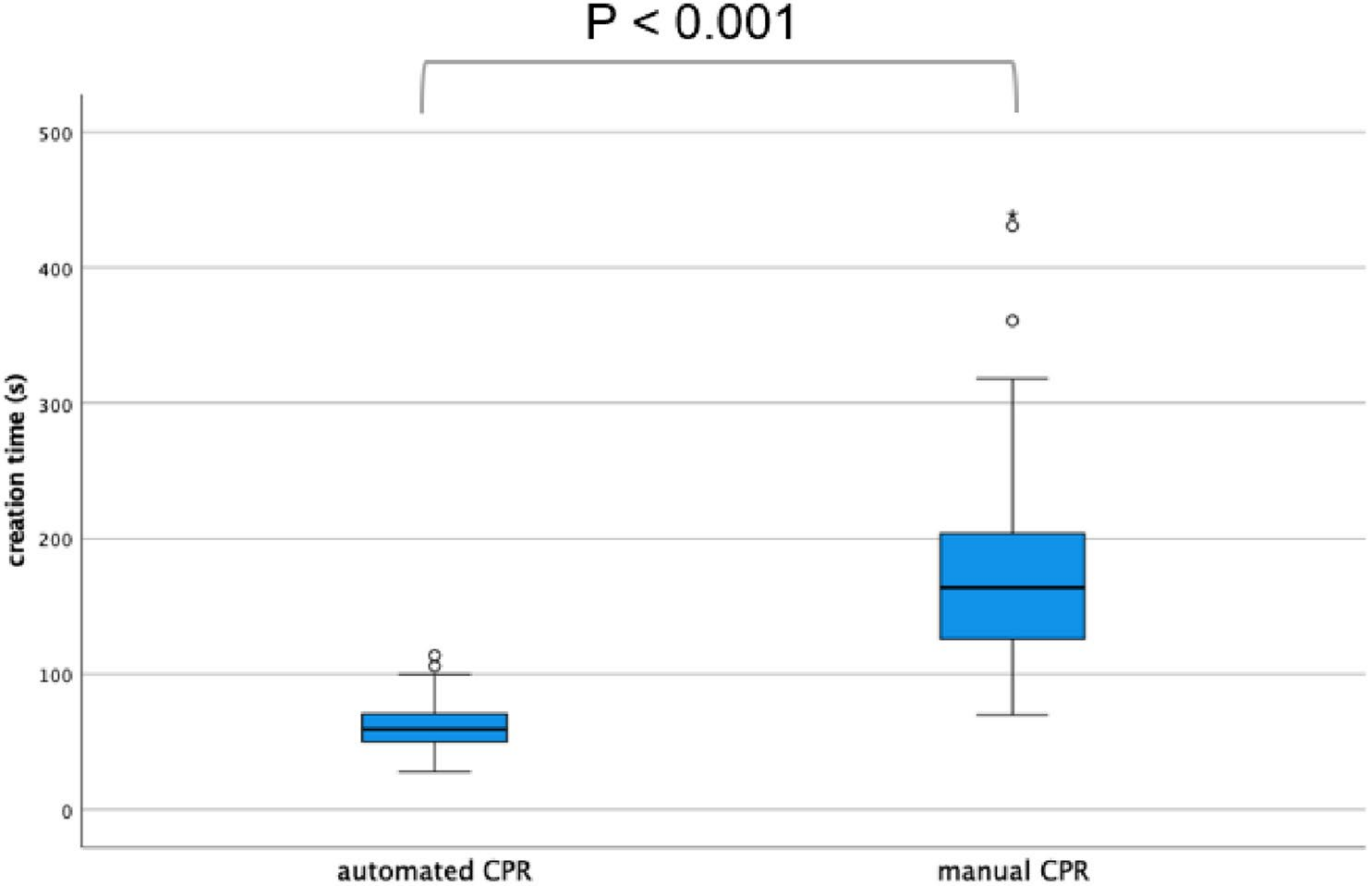
Time required to generate automated and manual CPR images. The solid line represents the median. Automated CPR images require significantly shorter times to be generated. *CPR* curved planar reconstruction

The mean time to create automated and manually created CPR images was 61.7 s (range: 28–114 s) and 174.6 s (range: 70–439 s), respectively. The required time was significantly shorter for the automated CPR group (*P* < 0.001) (Fig. 4).

### Total MPD length

**Fig. 5:**
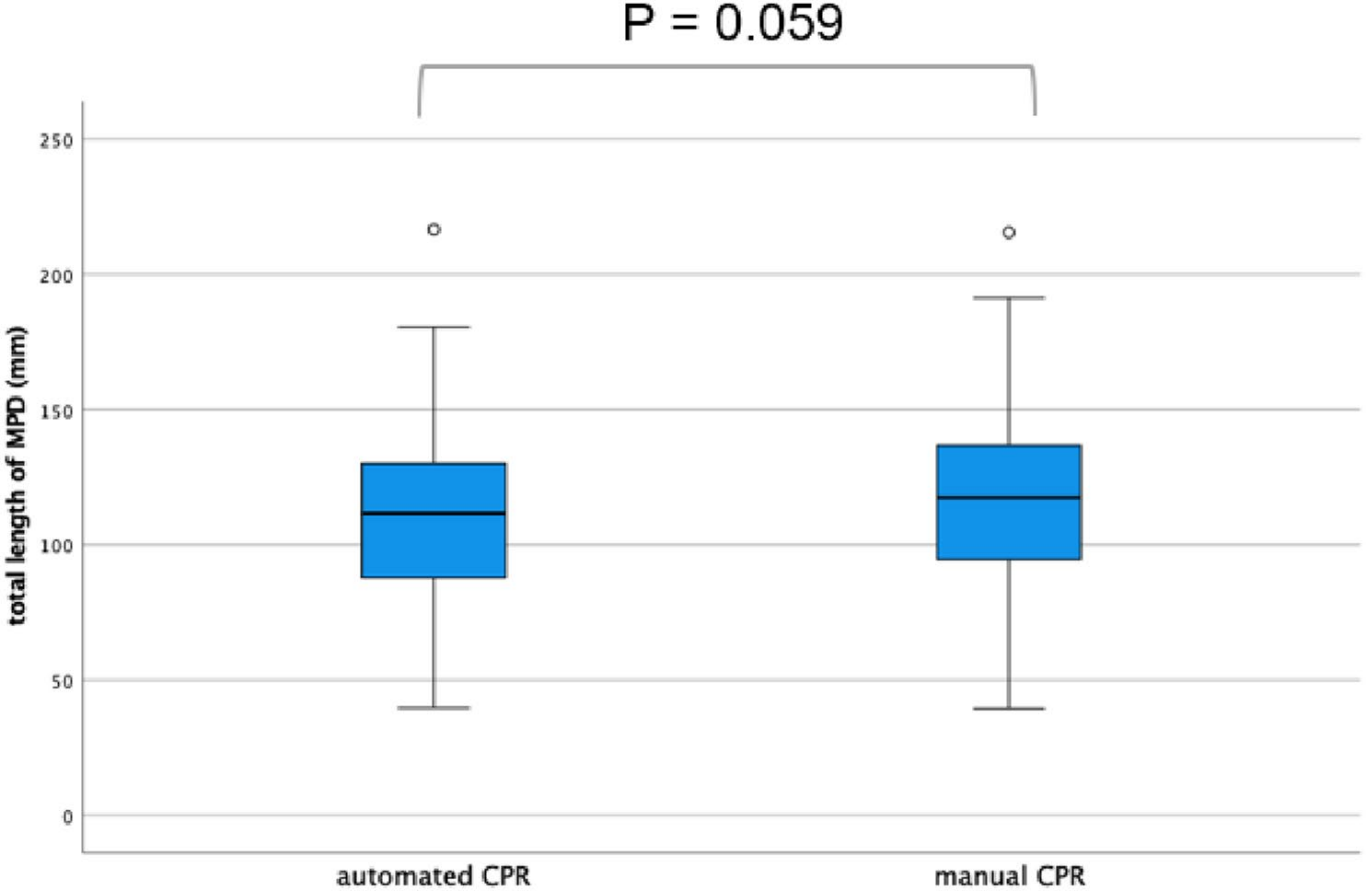
Total length of the MPD identified via automated and manual CPR images. The solid line represents the median. No significant differences were identified between the two groups. *MPD* main pancreatic duct, *CPR* curved planar reconstruction

The mean lengths of the MPD in the automated and manual CPR images were 110.5 and 115.6 mm, respectively. Although the total length appeared to be slightly longer in the manual CPR group, there was no statistically significant difference between the two groups (*P* = 0.059) (Fig. 5).

## Discussion

We evaluated the image quality and reconstruction time of automated CPR images in clinical settings. The mean creation time of the automated CPR images was 61.7 s, which was significantly shorter than that of the manual method. Moreover, in the subjective image analysis, the image quality of the automated CPR images was equivalent to that of the manually generated images, which included *κ* values of approximately 0.8.

There were five cases in which the image quality of the automated CPR images was inferior to that of the manual images. Although no certain trend could be found regarding the region in which the MPD was not detected, undetectable MPDs in these five cases were non-dilated (≤ 2 mm). This may have led to the result of a slightly shorter median length of the MPD compared with the manual CPR group, although the difference was not statistically significant. Hence, there is still room for improvement in the detection of non-dilated thin MPDs.

The generation time of automated CPR images was significantly shorter than that of manually generated images. The mean time required to generate CPR images manually was 174.6 s, which was shorter than that previously reported [5]. Advances in CT technology may be one of the contributing factors.

Although CPR images along the MPD have been reported to be useful for detecting pancreatic abnormalities [3–6], they are not used worldwide. It is assumed that there could have been two reasons: lack of experienced human resources and the time-consuming nature of the procedure. Our software might resolve these issues and assist in making high-quality CPR images more widely available and increasing the chances of improving the detectability of MPD abnormalities.

Our study has several limitations. We could not compare pancreatic lesion detectability between automated and manually created CPR images. The software performance was not fully evaluated because cases without MPD dilatation were not included. However, most of the situations for which CPR images are needed are cases of MPD dilatation. The study sample size was relatively small. Moreover, this was a single-institution retrospective study and only two readers evaluated the images. Therefore, the results might have differed from those obtained from a larger series.

In conclusion, our findings demonstrated that the software evaluated in the present study for the generation of CPR images along the MPD significantly reduced generation time compared with that of manually generated CPR images, without sacrificing image quality.
